# Effect of Nitrogen on Microbial Communities of Purple Mudstone Weathering Products in Southwest China: A Column Experiment

**DOI:** 10.3390/microorganisms12081548

**Published:** 2024-07-29

**Authors:** Chunpei Li, Wanting Li, Peng Xu, Xuan Wang, Jialiang Tang, Gangcai Liu, Ting Wang, Jixia Zhao

**Affiliations:** 1 College of Resources and Environment, Yunnan Agricultural University, Kunming 650201, China; 2 Key Laboratory of Mountain Surface Processes and Ecological Regulation, Institute of Mountain Hazards and Environment, Chinese Academy of Sciences, and Ministry of Water Conservancy, Chengdu 610041, Chinajltang@imde.ac.cn (J.T.); liugc@imde.ac.cn (G.L.); 3 University of Chinese Academy of Sciences, Beijing 100049, China

**Keywords:** nitrogen addition, purple mudstone weathering products, soil microbial community, co-occurrence network

## Abstract

Nitrogen application significantly affects microorganisms in agricultural ecosystems. However, it is still unclear how nitrogen application affects soil chemical properties and microbial communities in purple mudstone weathering products. In this study, a field soil column experiment was conducted in a typical purple soil area with four nitrogen fertilizer application gradients of 0 [CK], 280 [N1], 560 [N2], and 840 [N3] N kg ha^−1^. Nitrogen addition decreased the bacterial chao1 value and increased the bacterial evenness index. For both α- and β-diversity, the effect of nitrogen addition on bacteria was much greater than that on fungi. Nitrogen addition significantly increased the relative abundance of *Proteobacteria*, *Gemmatimonadetes*, *Bacteroidetes*, and *Ascomycota* and decreased the relative abundance of *Actinobacteria*, *Cyanobacteria*, and *Basidiomycota*. Both pH and TC are the most important soil chemical properties influencing the bacterial and fungal communities. With the increases in the nitrogen application rate, the co-occurrence network complexity increased and then decreased. In summary, nitrogen fertilizer application could significantly change the soil chemical properties, microbial community diversity, composition, and co-occurrence network of purple mudstone weathering products. Among them, the N2 treatment (560 N kg∙ha^−1^) can more effectively stimulate the soil nutrients, enhance microbial network complexity, and promote further weathering of purple mudstone.

## 1. Introduction

Soil microorganisms are vital to the soil ecosystem’s health and function [1,2]. Soil microbial community structure and diversity are affected by different factors, such as fertilization measures, soil physicochemical properties, and the interrelation of microbial species [3,4,5]. Nitrogen is essential for life systems, and the input of nitrogen fertilizer into agricultural systems is an important guarantee for crop production [6]. Nitrogen application may directly influence bacterial diversity by raising the soil ammonium level, whereas it may indirectly influence the bacterial community by decreasing soil pH [7]. Soil physicochemical properties such as soil organic matter and oxygen availability are considered to be the major drivers affecting soil microbial structure and activity [8]. In addition, microbial trophic interactions, consisting mainly of mutualism, predation, and parasitism, may present a diverse, complex, and stable soil microbiota [9]. Nitrogen application can change soil ecosystem function by affecting soil environment and microbial interactions.

In recent years, molecular ecological network analysis has been widely used in soil microbial ecology, as it can visually reflect the interactions between species in a biotic community [10]. The soil microbial co-occurrence network can be strongly affected by human activities, for instance, fertilizer application, irrigation, and tillage [11,12,13]. Nitrogen fertilizer application into soil affects soil chemical properties (i.e., pH and nutrient availability), which may induce changes in soil microbial interactions, resulting in transitions in the molecular ecological networks of microbial communities [14,15]. Excess nitrogen fertilizer application breaks the interdependence of bacterial and fungal species and weakens competition, affecting ecological network relationships [16]. The dominant microorganisms in molecular ecological networks are affected after nitrogen fertilizer application. *Proteobacteria*, *Actinobacteria*, and *Ascomycota* are the dominant microorganisms in nitrogen fertilizer application [17]. Therefore, nitrogen application can alter the co-occurrence network of bacteria–fungi–soil properties and change their interactions by changing the soil environment.

As classified by the U.S. Department of Agriculture, purple soil is formed of purple rocks (mudstones and/or sandstones) or their weathering products [18]. Crops can be planted directly in the purple rock fragments or gravels, and particles <2 mm in weathered products are commonly viewed as soil particles [19]. Purple rocks are widely distributed in the humid subtropical climate region, characterized by serious soil erosion and a shallow layer of purple soil, which result in intensive fertilizer application to maintain and improve crop yield in this area. The purple mudstone of the Penglai group (J_3_p) is one of the most widely distributed alkaline purple parent rocks in Sichuan Basin, China, and the exposed surface area of J_3_p purple mudstone accounts for 26.23% of the exposed surface area of total purple parent rocks in Sichuan Basin. The exposed purple rock is suggested to be affected by hydrothermal conditions, acid deposition, and fertilization [20,21], promoting the weathering process. Ammonium bicarbonate is the main fertilizer type applied in the study area, and nitrogen fertilizer amounts in wheat and maize seasons are 130 and 150 N kg ha^−1^ in this study area, respectively. However, the effects of different amounts of ammonium bicarbonate application on the microbial community characteristics, the co-occurrence network of microorganisms, and the chemical properties of purple mudstone weathering products are still unclear.

Therefore, to evaluate the effects of nitrogen fertilizer application levels on soil chemical properties, microbial communities, and the interaction between soil chemical properties and microbial communities of purple mudstone weathering products, a simulated soil column experiment was established in a typical purple soil area in Sichuan Province. Considering the changes in soil chemical properties in the soil microenvironment by nitrogen fertilizers and the difference in microbial species for nutrition bioavailability, this study hypothesized that (1) soil chemical properties are significantly altered by different nitrogen fertilizer applications, which shift the diversity and composition of soil microbial communities, and that (2) different nitrogen fertilizer applications affect the co-occurrence network of soil environment–bacteria–fungi by changing soil chemical properties.

## 2. Materials and Methods

### 2.1. Study Sites

The field soil column experiment was carried out at the Yanting Purple Soil Agricultural Ecological Experiment Station, Chinese Academy of Sciences (31°16′26.20″ N, 105°27′27.20″ E). In this region, the mean annual rainfall and long-term annual average temperature are 826 mm and 17.3 °C, respectively. The study region belongs to the central subtropical humid climate zone. Most precipitation occurs from May to September.

### 2.2. Experiment Design and Sampling

The effect of fertilization on rock chemical weathering is a very long process. In order to amplify the effect of nitrogen fertilizer application on the soil properties and microbial communities of purple soil weathering products, four nitrogen addition levels (0, 280, 560, and 840 N kg∙ha^−1^ described as CK, N1, N2, and N3) were set up. 280 N kg∙ha^−1^ is the conventional nitrogen application level for purple soil in the Sichuan Basin. The field soil column experiment began in October 2021 with three repetitions of each. Before the start of the experiment, the fresh purple rocks were transported to the laboratory and air-dried at ambient temperature. J_3_p purple mudstone of the air-dried rocks was screened for particle sizes of >60, 40 to 60, 20 to 40, 10 to 20, and 5 to 10 mm. The purple mudstone of the respective particle size after screening was employed in this experiment. A schematic plot of the soil column is shown in Figure S1, and the preparation steps were as follows: (I) A circular PVC tube with a diameter of 160 mm and a height of 400 mm was employed to form a soil column, the tube cap was connected to the bottom of the soil column, and a small hole of 10 mm was opened in the center of the pipe cap to simulate the infiltration process of natural soil solution. (II) At the bottom of the soil column, two layers of nylon net with a layer of asbestos between them were placed as isolation net. (III) The purple rock particles were loaded into the soil column from the bottom to the top in the order of >60, 40 to 60, 20 to 40, 10 to 20, and 5 to 10 mm. The proportion of each particle size was its mass proportion in the original J_3_p purple mudstone, and the total mass of the purple rock particles loaded into the soil column was controlled to be 600 g. (IV) Different amounts of nitrogen fertilizer were dissolved in deionized water and added to the corresponding treated soil columns. The basic chemical composition of J_3_p purple mudstone is shown in Table S1.

In October 2022, soil samples were randomly collected from each treatment. After sampling, the soil samples were sieved (2 mm) and divided into three subsamples. One subsample was air-dried to analyze soil chemical properties, and the other was stored at −80 °C to analyze soil microbial communities.

### 2.3. Measurement of Soil Chemical Properties

The determination methods of soil chemical properties are as follows [22]. Soil pH was measured with a pH meter (PE-10, Sartorius, Göttingen, Germany) using a 1:2.5 (*w*/*v*) soil-to-water ratio. Soil total carbon content (TC) was analyzed by oxidizing organic C with potassium dichromate (K_2_Cr_2_O_7_). Soil total nitrogen content (TN) was measured using the Kjeldahl method. After the samples were wet-digested with sulfuric acid (H_2_SO_4_) and perchloric acid (HClO_4_), soil total phosphorus (TP) and total potassium (TK) were measured using the spectrophotometry and flame photometry methods, respectively. Soil alkaline nitrogen (AN) was determined using the alkaline diffusion method. Soil available phosphorus (AP) was measured using the molybdenum-blue method after extraction with sodium bicarbonate (NaHCO_3_). Soil available potassium (AK) was measured using the flame photometry method after extraction with ammonium acetate (CH_3_COONH_4_).

### 2.4. DNA Extraction and Sequencing

The DNA was extracted from 0.5 g of weathering products of J_3_p purple mudstone using a Fast DNA SPIN extraction kit (MP Biomedicals, Santa Ana, CA, USA) following the manufacturer’s protocol. The quantity and quality of DNA extracts were detected using a Nanodrop ND-1000 UV–visible spectrophotometer (Thermo Fisher Scientific, Waltham, MA, USA) and agarose gel electrophoresis, respectively. The amplification of the bacterial 16S rRNA gene was performed using primer sets 338F/806R, and the fungal ITS region was amplified using primer sets ITS5/ITS2 (Table S2). The amplification system (25 μL) was as follows: 5 μL reaction buffer (5×), 5 μL GC buffer (5×), 2 μL dNTP (2.5 mM), 1 μL forward and reverse primers (10 μM), 2 μL DNA template, 0.25 μL Q5 DNA polymerase, and 8.75 μL double-distilled H_2_O to a final volume of 25 μL. The polymerase chain reaction (PCR) protocol was performed in triplicate using the following conditions: 2 min at 98 °C for initial denaturing, followed by 25 to 30 cycles of 98 °C for 15 s, 55 °C for 30 s, 72 °C for 30 s, 72 °C for 5 min, and 10 °C for hold. PCR amplicons were purified with Agencourt AMPure Beads (Beckman Coulter, Indianapolis, IN, USA) and quantified using the PicoGreen dsDNA Assay Kit (Invitrogen, Carlsbad, CA, USA). After the quantification step, the 16S rRNA and ITS gene fragments were sequenced using the Novaseq-PE250 pattern of the Illumina MiSeq platform. The raw amplicon sequences were deposited in the Sequence Read Archive and assigned the following BioProject accession number: PRJNA990361.

The paired-end reads were assembled using FLASH version 1.2.11 to obtain raw tags. The resulting FASTQ files were generated using the DADA2 plugin in the QIIME 2 program [23]. The DADA 2 denoised sequences are known as amplicon sequence variants (ASVs), and their taxonomic assignment was performed using the vsearch consensus taxonomy classifier using QIIME 2.

### 2.5. Statistical Analysis

Before analyses, the normality and homogeneity of the variables were checked using the Kolmogorov–Smirnov and Levene tests, respectively. One-way analysis of variance (ANOVA), followed by Duncan’s post hoc test (*p* < 0.05), was performed to compare differences in soil chemical properties, α-diversity, microbial relative abundance, and co-occurrence network properties. Microbial α-diversity included Chao1, evenness, and Shannon indexes. The relative abundance of the microbial community composition at the phylum level was counted to explore the dominant phyla under different nitrogen fertilizer applications, and a graph of microbial community composition at the phylum level was drawn using Origin 2021. To analyze the difference in microbial β-diversity under different treatments, principal coordinate analysis (PCoA) was used (the method of weighted UniFrac distance). To explore the effects of soil chemical properties on bacteria and fungi, redundancy analysis (RDA) was calculated based on the “vegan” package of R version 4.3.0, the importance of each chemical index was calculated for microbial communities using PERMANOVA, and variance partitioning analysis (VPA) was carried out to understand the contribution nitrogen fertilizer levels and soil chemical properties to microbial community based on the RDA results [24]. The co-occurrence network of bacteria, fungi, and soil chemical properties was calculated using the “ggClusterNet” R package [25], and co-occurrence network properties were displayed. PCoA, RDA, VPA, and co-occurrence network analyses were performed using R version 4.3.0. Moreover, the analyses related to α-diversity, PCoA, RDA, VPA, and co-occurrence network were calculated based on the ASV level, and composition was performed at the phylum level.

## 3. Results

### 3.1. Soil Chemical Properties

With the addition of nitrogen fertilizer, the pH, TK, and AN of purple mudstone weathering products increased and reached the highest values under the N2 treatment, and then decreased, whereas the TC of purple mudstone weathering products showed the opposite trend. Furthermore, the contents of TP and AP in the purple mudstone weathering products increased, and AK decreased from the N1 to the N3 treatment. Compared to the CK treatment, the pH, TN, TK, and AN after nitrogen fertilizer application increased by 3.41% to 3.91%, 2.72% to 21.74%, 0.53% to 2.69%, and 74.04% to 245.19%, respectively, but the TC after nitrogen fertilizer application decreased by 32.78% to 68.05%. Moreover, compared with CK, the TP in the N1 and N2 treatments decreased by 6.07% and 3.64%, while that in the N3 treatment increased by 8.07%. The AP under the N1 and N2 treatments decreased by 51.28% and 25.64% compared to CK, respectively, but the AP under the N3 treatment was enhanced by 84.62%. The AK under the N1 treatment increased by 4.76%, but the AK under the N2 and N3 treatments decreased by 4.76% and 9.52% compared to CK, respectively (Table 1).

### 3.2. Diversity of Soil Microbial Communities

In total, there were 97,280 to 136,508 raw bacterial 16S rRNA sequences and 168,295 to 199,517 raw fungal ITS sequences across all samples. After optimized sequence extraction, 68,947 to 100,116 sequences were clustered into 50,627 bacterial ASVs and 105,922 to 157,341 sequences into 2043 fungal ASVs.

**Figure 1:**
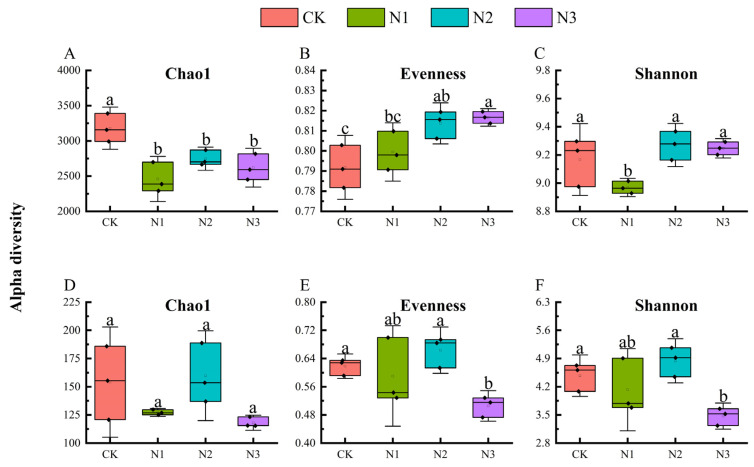
Chao1 richness, evenness, and Shannon indices for bacterial (**A**–**C**) and fungal (**D**–**F**) communities in N fertilizer treatments (mean ± SE). CK, no fertilizer control; N1, 280 N kg ha^−1^; N2, 560 N kg ha^−1^; N3, 840 N kg ha^−1^. Different letters indicate significant differences among treatments.

The α-diversity of soil bacterial communities varied with nitrogen fertilizer treatment. Compared to the CK treatment, the Chao1 index (13.64–22.66%) and Shannon index (N1, 2.17%) of bacterial communities were decreased, whereas the Shannon index (N2, 1.12%; N3, 0.87%) and evenness index (0.03–0.24%) of bacterial communities were enhanced (Figure 1A–C). In addition, the α diversity of fungal communities was relatively discrete among biological replicates. The α-diversity (including Chao1, evenness, and Shannon indices) of fungal communities under the N2 treatment increased by 3.69%, 8.28%, and 2.43%, whereas the α-diversity (including Chao1, evenness, and Shannon indices) of fungal communities under the N1 and N3 treatments decreased by 17.41% to 23.37%, 7.80% to 22.43%, and 3.28% to 15.70%, respectively (Figure 1D–F). With the increase in nitrogen addition from N1 to N3, the α-diversity indices of bacterial and fungal communities showed a tendency to rise and then fall, except for the bacterial evenness index, which showed a consistent increase (Figure 1).

**Figure 2:**
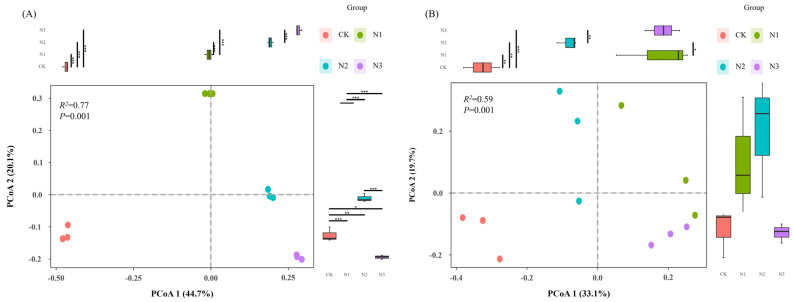
PCoA ordination of bacterial (**A**) and fungal (**B**) communities in N fertilizer treatments based on weighted UniFrac distance. CK, no fertilizer control; N1, 280 N kg ha^−1^; N2, 560 N kg ha^−1^; N3, 840 N kg ha^−1^. *, ** and *** represent significant difference with *p* values < 0.5, 0.01 and 0.001, respectively.

The β-diversity of microbial communities formed four clusters by the PCoA of weighted UniFrac distance and changed strongly after nitrogen fertilizer application (Figure 2). The explanatory degree of nitrogen fertilizer levels on the β-diversity of bacterial and fungal communities was calculated as 64.8% and 52.80%, respectively. More specifically, the explanatory degree of the first and second axes of bacterial β-diversity was calculated as 44.70% and 20.10%, and the explanatory degree of the first and second axes of fungal β-diversity was calculated as 33.10% and 19.70%, respectively. Furthermore, with increasing nitrogen fertilizer levels, bacterial β-diversity showed an increasing trend, but an opposite trend in fungal β-diversity was measured (Figure S2).

### 3.3. Composition of Soil Microbial Communities

**Figure 3:**
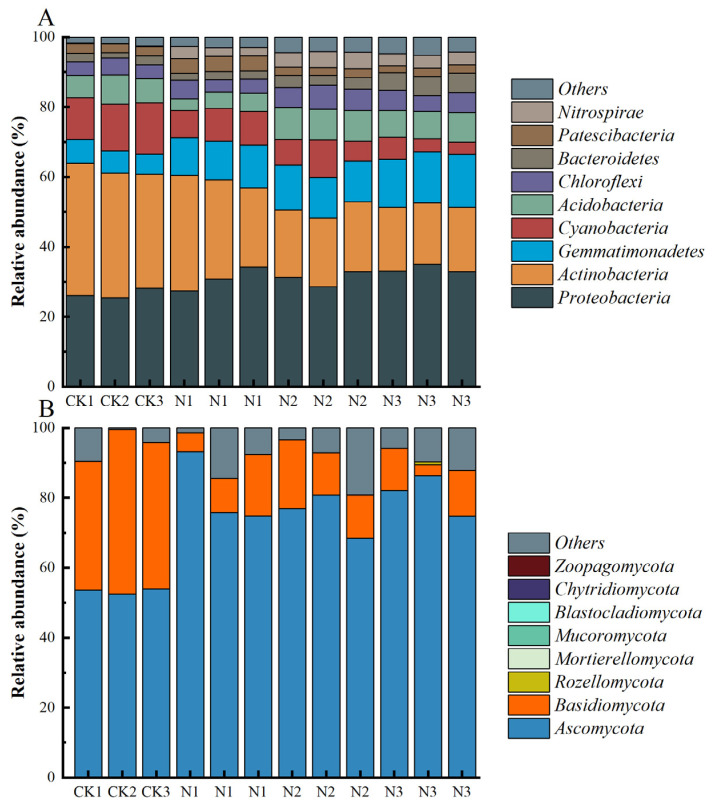
Taxonomic composition of soil bacterial (**A**) and fungal (**B**) communities at the phylum level in N fertilizer treatments. The top nine bacterial phyla and the top eight fungal phyla with the highest relative abundance are presented in the stacked column charts. CK, no fertilizer control; N1, 280 N kg ha^−1^; N2, 560 N kg ha^−1^; N3, 840 N kg ha^−1^.

Nitrogen addition significantly affected the structure of bacterial and fungal communities in the purple mudstone weathering products. At the phylum level, *Proteobacteria* (30.47%), *Actinobacteria* (25.28%), *Gemmatimonadetes* (11.08%), *Ascomycota* (72.77%), and *Basidiomycota* (19.23%) were determined as the predominant phyla of microbial communities (Figure 3A,B). Specifically, compared to the CK treatment, the relative abundance of *Proteobacteria* (16.00–26.65%), *Gemmatimonadetes* (79.55–129.33%), *Acidobacteria* (N2, 24.03%; N3, 10.76%), *Chloroflexi* (0.40–46.82%), *Bacteroidetes* (0.23–146.18%), *Patescibacteria* (N1, 68.89%), and *Nitrospirae* (14.20–23.72 times) among bacterial communities and *Ascomycota* (41.30–52.33%) among fungal communities was significantly enhanced, whereas the relative abundance of *Actinobacteria* (20.74–48.72%), *Cyanobacteria* (32.80–66.47%), *Acidobacteria* (N1, 39.02%), and *Patescibacteria* (N2, 8.85%; N3, 12.15%) among bacterial communities and *Basidiomycota* (64.98–77.41%) among fungal communities significantly declined (Figure S3). With increasing nitrogen fertilizer levels, the relative abundance of *Proteobacteria*, *Gemmatimonadetes*, and *Bacteroidetes* showed an increasing trend, but the relative abundance of *Actinobacteria*, *Cyanobacteria*, and *Patescibacteria* showed the opposite trend. With increasing nitrogen fertilizer levels, the relative abundance of *Acidobacteria*, *Chloroflexi*, *Nitrospirae*, and *Basidiomycota* showed increasing and then decreasing trends, but *Basidiomycota* showed the opposite trend (Figure S3).

### 3.4. Relationship between Soil Chemical Properties and Soil Microbial Communities

**Figure 4:**
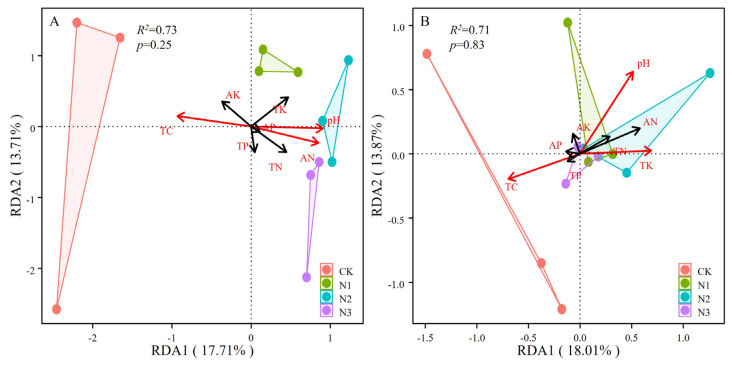
Relationships between the soil chemical properties and the bacterial (**A**) and fungal (**B**) communities. CK, no fertilizer control; N1, 280 N kg ha^−1^; N2, 560 N kg ha^−1^; N3, 840 N kg ha^−1^.

**Figure 5:**
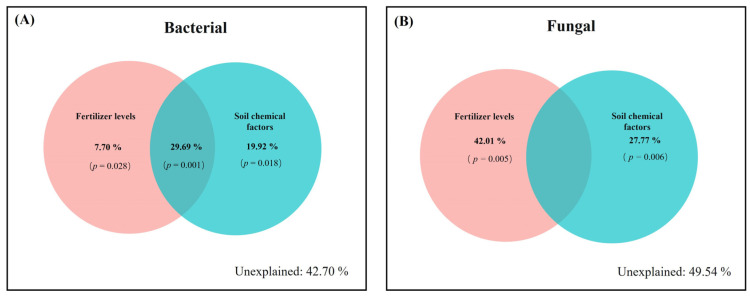
VPA of the effects of fertilizer levels and soil chemical factors on bacterial (**A**) and fungal (**B**) communities. In the bacterial community, soil chemical factors included pH, TC, and AN. In the fungal community, soil chemical factors included pH, TC, and TK.

The relationships between soil microbial communities and soil chemical properties were analyzed using RDA (Figure 4). The top two RDA factors explained 31.42% of community variation in bacterial communities and 31.88% in fungal communities, and the key chemical properties of purple mudstone weathering products for bacterial communities (pH, *R*^2^ = 0.82, *p* < 0.05; TC, *R*^2^ = 0.88, *p* < 0.01; AN, *R*^2^ = 0.76, *p* < 0.01) and fungal communities (pH, *R*^2^ = 0.56, *p* < 0.05; TC, *R*^2^ = 0.46, *p* < 0.05; TK, *R*^2^ = 0.47, *p* < 0.05) were calculated using the post hoc test of PERMANOVA (Table S3). Meanwhile, RDA results also confirmed that the effects of the chemical properties of purple mudstone weathering products on bacterial communities (*R*^2^ = 0.73) were slightly greater than those on fungal communities (*R*^2^ = 0.71) in this study (Figure 4). Furthermore, the VPA indicated that nitrogen fertilizer application explained 57.30% of the variation in bacterial communities, including fertilizer levels (7.70%) and soil chemical properties (19.92%) (Figure 5A), and 50.46% of the variation in fungal communities, including fertilizer levels (42.01%) and soil chemical properties (27.77%) (Figure 5B).

**Figure 6:**
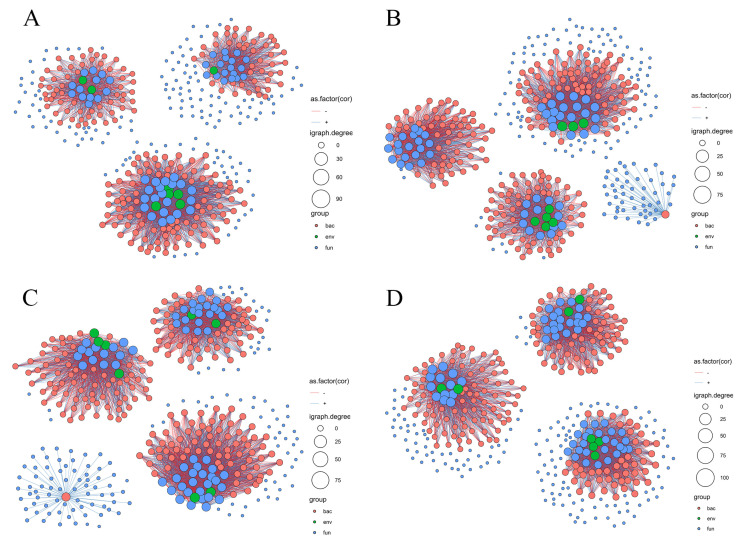
Co-occurrence network analysis of bacterial communities, fungal communities, and the environment in purple mudstone weathering products under CK (**A**), N1 (**B**), N2 (**C**), and N3 (**D**) treatments. Orange, green, and blue circles indicate bacteria, soil chemical properties, and fungi, respectively. The size of the circle indicates the importance of bacteria, soil chemical properties, and fungi. Orange and blue lines indicate the positive and negative effects of the co-occurrence network, respectively.

**Figure 7:**
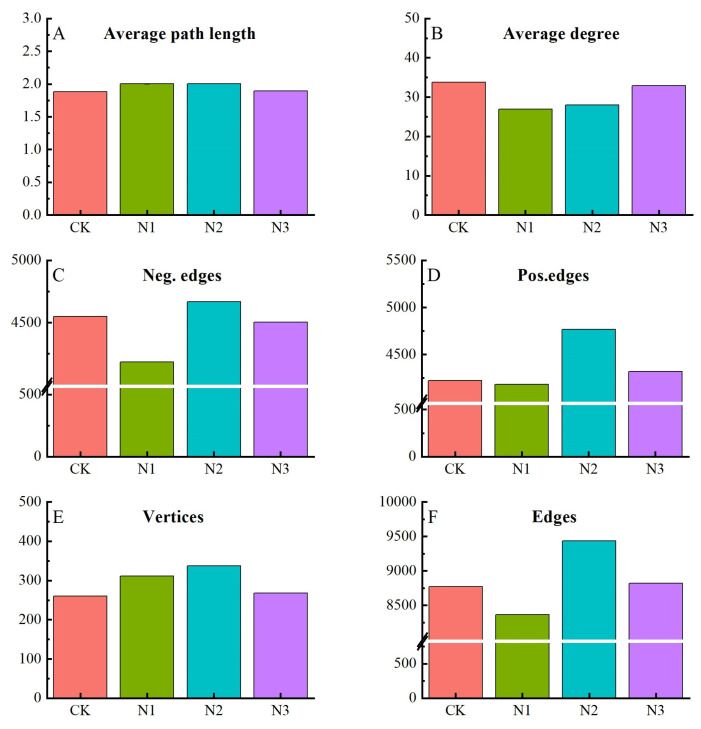
Characteristics of microbial co-occurrence networks. Average path length (**A**), average degree (**B**), negative edges (**C**), positive edges (**D**), vertices (**E**), and edges (**F**). CK, no fertilizer control; N1, 280 N kg ha^−1^; N2, 560 N kg ha^−1^; N3, 840 N kg ha^−1^.

Compared to the CK treatment, the edges (N1, 4.61%), average degree (2.41–20.25%), positive edges (N1, 0.95%) and negative edges (N1, 8.00%; N3, 1.01%) of the co-occurrence network after nitrogen fertilizer application were reduced, whereas the average path length (0.60–6.66%), edges (N2, 7.57%; N3, 0.59%), positive edges (N2, 12.88%; N3, 2.32%), negative edges (N2, 2.64%), and vertices (3.08–29.62%) of the co-occurrence network after nitrogen fertilizer application were enhanced (Figure 6 and Figure 7). With increasing nitrogen fertilizer levels, the average path length, negative edges, positive edges, vertices, and edges of the co-occurrence network showed increasing and then decreasing trends, and the average edges and nitrogen fertilizer levels showed a positive correlation in this study, confirming that the co-occurrence network stability and complexity were significantly altered (Figure 6 and Figure 7).

## 4. Discussion

### 4.1. Effects of N Fertilizer Application on Soil Chemical Properties

This study found that the soil chemical properties of purple mudstone weathering products were significantly affected by nitrogen addition (Table 1). The soil pH, TK, and AN of purple mudstone weathering products were enhanced, but the TC was reduced after nitrogen fertilizer application. Nitrogen fertilizer retains hydrogen protons (H^+^) in the soil, leading to decreased pH [26]. However, the hydrolysis process of ammonium bicarbonate induces the formation of an alkaline microenvironment, resulting in an increase in soil pH after nitrogen application [27]. The results of this study are consistent with previous studies that the isomorphous substitution of NH_4_^+^ produced by the hydrolytic process of ammonium bicarbonate with K^+^ of clay minerals is the main reason for the increase in the TK of the purple mudstone weathering products in this study. Nitrogen mineralization was enhanced by nitrogen fertilizer application, promoting effective nitrogen release (Wang et al., 2023), which has been extensively studied and well understood [28]. Nitrogen application strongly altered soil C and N contents and accelerated the decomposition process of C by increasing N availability, which was the main reason for the reduction in TC in purple mudstone weathering products [29]. In conclusion, the N2 treatment was the optimal nitrogen application level in this study when only the nutrient characteristics of purple mudstone weathering products were considered, while the N1 treatment was the optimal nitrogen application level in this study when soil carbon storage was considered.

### 4.2. Effects of N Fertilizer Application on Microbial Diversity and Composition

Consistent with the hypothesis, nitrogen addition significantly altered the soil microbial diversity of purple mudstone weathering products (Figure 1 and Figure 2). Nitrogen addition significantly reduced the Chao1 index in bacterial α-diversity and reduced the number of ASVs occurring once in the bacterial community. This is consistent with previous results showing that nitrogen addition directly reduced the soil bacterial Chao1 index by increasing soil available nitrogen [30]. In contrast to the Chao1 index, nitrogen addition significantly increased the evenness index of the soil bacterial α-diversity. Nitrogen addition broke the interactions between different species and drove the bacterial community toward homogenization. Bacterial and fungal communities had different responses to nitrogen addition. The response of the fungal α-diversity index to nitrogen addition was significantly lower than that of bacteria, and the principal coordinate analysis of fungi was more discrete than that of bacteria. Fungal communities presented higher adaptability to nitrogen addition. The Shannon index of microbial communities was highest under the N2 treatment for both bacteria and fungi, which is similar to the results of previous studies. Nitrogen addition significantly increased the soil microorganisms’ Shannon–Weiner index, and excessive nitrogen addition significantly inhibited Shannon diversity [31].

Nitrogen application significantly altered the microbial community composition of purple mudstone weathering products. According to the eutrophic–oligotrophic concept, eutrophic microorganisms usually have a high growth rate under nutrient-rich conditions, while oligotrophic microorganisms are able to maintain growth under nutrient-poor conditions [32]. *Proteobacteria* microbes are eutrophic bacteria that grow rapidly after nitrogen addition, which is consistent with the results of a metal analysis about the effect of long-term nitrogen application on bacterial community composition [33]. In total, 72.77% of soil fungal ASVs belonged to *Ascomycota*. *Ascomycota* are sensitive to fertilizer treatment, and high nitrogen input in fertilizer management changed the C/N ratio in the purple mudstone weathering products, which created a good living environment for *Ascomycota* microorganisms. *Cyanobacteria* have the ability to fix N2 from the atmosphere, which is important for replenishing soil N pools [34].

### 4.3. Relationship of Soil Chemical Properties and Microbial Community

Soil properties interact with microbial communities. Microorganisms participate in the transformation of soil organic matter and nutrients, and can change the soil environment through a variety of physical, chemical, and biological mechanisms. In turn, such microbiota-mediated changes in soil properties can affect microbial community structure and function [8]. A large number of studies have assessed the effects of pH on microbial communities. In this study, RDA showed that pH was the most important soil chemical property affecting soil bacteria and fungi. One possible explanation is that pH has a physiological constraint on soil microorganisms and plays an important role in membrane-bound proton pump stabilization [35]. Another explanation is that soil pH indirectly affects the microbial community by altering soil carbon and nutrient availability [36]. In addition, bacteria and fungi have different tolerance ranges to pH; bacteria have a narrower tolerance range to pH, and such a conclusion is also supported by our results. It has been proven that mineral–microbial interactions can promote rock weathering and soil formation, and enhance the cycling of carbon and nutrients in soil [37]. Soil TC was significantly correlated with the changes in soil bacterial and fungal communities [38]. The interaction between purple mudstone and microorganisms affected the soil carbon pool.

In this study, the co-occurrence network complexity of the N2 treatment was the highest. Previous studies have shown that the complexity of microbial co-occurrence networks is controlled by C and N content [39]; interactions within microbial communities are another cause of microbial community alteration, and the results of this study can confirm these ideas. In short, N fertilizer significantly altered soil chemical properties and altered bacterial and fungal interactions. In addition, the number of connections between bacteria and soil chemical properties in the co-occurrence network was higher than that between fungi and soil chemical properties, and the number of connections decreased with increasing fertilizer levels. Previous studies have shown that bacteria are more sensitive to soil chemical properties than fungi [40], and higher NH_4_^+^-N content inhibits bacterial growth, but cannot affect fungal growth through changes in soil NH_4_^+^-N content or pH [41], and previous studies support the findings of this study. Nitrogen fertilizer affects soil chemical properties, leading to changes in microbial community composition, diversity, and phylum interactions, which in turn alter co-occurrence network properties.

## 5. Conclusions

This study revealed the characteristics of soil microbial communities and soil chemical drivers of purple mudstone weathering products under different nitrogen fertilizer levels. The pH, TK, and AN content in the purple mudstone weathering products increased and TC decreased with increasing N fertilizer application rates. Nitrogen addition decreased the chao1 index of bacteria and increased their evenness index. Compared with fungi, bacteria were more sensitive to nitrogen addition. The relative abundance of the eutrophic *Proteobacteria* and *Cyanobacteria* increased with increasing N addition. Furthermore, the explanatory degree of key soil chemical properties for bacterial (pH, TC, and AN) and fungal (pH, TC, and TK) communities was calculated as 30.88% and 31.67%, respectively. With the increase in the nitrogen application rate, the co-occurrence network complexity increased and then decreased. Nitrogen fertilizer affected soil microbial community diversity, composition, and co-occurrence network by affecting soil chemical properties, especially pH, carbon, and nitrogen contents. The N2 treatment (560 N kg∙ha^−1^) was more effective than other treatments in stimulating soil nutrients and enhancing the complexity of microbial network, which promoted the further weathering of purple mudstone.

## Figures and Tables

**Table 1:** Soil chemical properties of purple mudstone weathering products after N fertilizer application (mean ± SE). CK, no fertilizer control; N1, 280 N kg ha^−1^; N2, 560 N kg ha^−1^; N3, 840 N kg ha^−1^. Different letters indicate significant differences among treatments.

Treatments	CK	N1	N2	N3
pH	7.92 ± 0.08 b	8.22 ± 0.01 a	8.23 ± 0.03 a	8.19 ± 0.02 a
TC (g kg^−1^)	2.41 ± 0.00 a	1.62 ± 0.11 b	0.77 ± 0.11 d	1.20 ± 0.10 c
TN (g kg^−1^)	1.84 ± 0.02 b	1.95 ± 0.08 b	1.89 ± 0.10 b	2.24 ± 0.09 a
TP (g kg^−1^)	11.53 ± 0.05 ab	10.83 ± 0.76 b	11.11 ± 0.03 ab	12.46 ± 0.35 a
TK (g kg^−1^)	18.65 ± 0.24 a	19.09 ± 0.14 a	19.17 ± 0.00 a	18.74 ± 0.14 a
AN (mg kg^−1^)	10.45 ± 0.00 c	18.11 ± 3.11 b	35.98 ± 0.99 a	35.41 ± 3.17 a
AP (mg kg^−1^)	0.39 ± 0.10 b	0.19 ± 0.07 c	0.29 ± 0.08 c	0.72 ± 0.17 a
AK (g kg^−1^)	0.21 ± 0.00 a	0.22 ± 0.00 a	0.20 ± 0.00 ab	0.19 ± 0.01 b

## Data Availability

For the soil microorganism data, the raw amplicon sequences were deposited in the Sequence Read Archive and assigned the following BioProject accession number: PRJNA990361.

## Supplementary Materials

The following supporting information can be downloaded at: https://www.mdpi.com/article/10.3390/microorganisms12081548/s1, Figure S1: A schematic plot of the soil column; Figure S2: The relationship between fertilization levels and beta diversity of microbial was calculated based on the linear regression analysis; Figure S3: Analysis of differences in the relative abundance of dominant bacterial phyla and fungal phyla; Table S1: Main chemical element content of the J3p purple mudstone; Table S2: Sequencing primers for microbial communities; Table S3: The chemical properties of purple mudstone weathering products were calculated by redundant analysis.
