# Supplementary material for: Effect of Nitrogen on Microbial Communities of Purple Mudstone Weathering Products in Southwest China: A Column Experiment
Source: Microorganisms. 2024 Jul 29;12(8):1548. doi: 10.3390/microorganisms12081548 (PMC11356197; doi:10.3390/microorganisms12081548)
Supplement: Supplementary file 1 [file microorganisms-12-01548-s001.zip › microorganisms-3113827-supplementary.pdf]

Effect of Nitrogen on Microbial Communities of Purple Mudstone Weathering Products in Southwest China: A Column Experiment

Chunpei Li[a,b,c, #], Wanting Li[a,#], Peng Xu[b], Xuan Wang[b,c], Xuemei Wang[d], Jialiang Tang [b], Gangcai Liu [b], Ting Wang [a,*], Jixia Zhao[a,*]

[a] *College of Resources and Environment, Yunnan Agricultural University, Kunming 650000, China;*

[b] *Key Laboratory of Mountain Surface Processes and Ecological Regulation, Chinese Academy of Sciences, Institute of Mountain Hazards and Environment, Chinese Academy of Sciences, and Ministry of Water Conservancy, Chengdu 610041, China;*

[c] *University of Chinese Academy of Sciences, Beijing 100049, China;*

[d] *School of Resource and Environmental Engineering, Mianyang Normal University, Mianyang 621000, Sichuan, China*

[#] These authors have contributed equally to this work and share first authorship

* These authors have contributed equally to this work and share corresponding author:

* Corresponding author:

Jixia Zhao

College of Resources and Environment, Yunnan Agricultural University

No.95, Heijin Road, Panlong District, Kunming, China

Post code: 650201

Tel: +86 0871-65227550

Fax: +86 0871-65227550

Email: zhaojixiacc@163.com

Table S1. Main chemical element content of the J_3p purple mudstone /%

SiO_2	Al_2O_3	Fe_2O_3	K_2O	Na_2O	CaO	MgO
54.79±2.64	17.88±1.41	4.28±0.11	3.42±0.01	2.23±0.00	16.32±0.12	1.08±0.09

Table S2. Sequencing primers for microbial communities

	Upstream primer	Reverse primer
Bacterial community	338F-ACTCCTACGGGAGGCAGCA	806R-GGACTACHVGGGTWTCTAAT
Fungal community	ITS5-GGAAGTAAAAGTCGTAACAAGG	ITS2-GCTGCGTTCTTCATCGATGC

Table S3. The chemical properties of purple mudstone weathering products were calculated by redundant analysis

Soil physicochemical factors	Bacterial community		Fungal community	
	R^2	p	R^2	p
pH	0.82	0.01	0.56	0.04
TC	0.88	0.00	0.46	0.04
TN	0.30	0.21	0.09	0.66
TP	0.09	0.59	0.02	0.86
TK	0.32	0.18	0.47	0.04
AN	0.76	0.00	0.33	0.14
AP	0.01	0.93	0.02	0.90
AK	0.20	0.33	0.03	0.82

Notes: TC, total carbon; TN, Total nitrogen; TP, total phosphorus; TK, total potassium; AN, alkaline nitrogen; AP, available phosphorus; AK, available potassium.

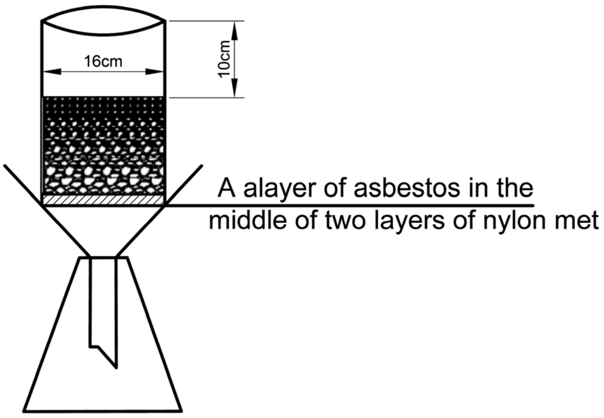

Figure S1. A schematic plot of the soil column.

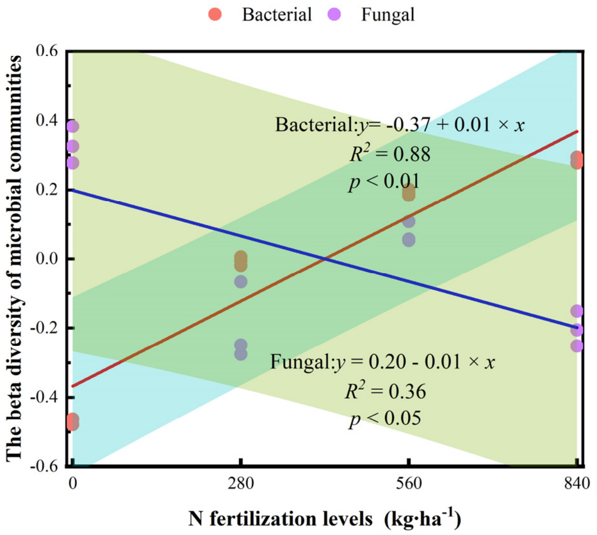

Figure S2. The relationship between fertilization levels and beta diversity of microbial was calculated based on the linear regression analysis.

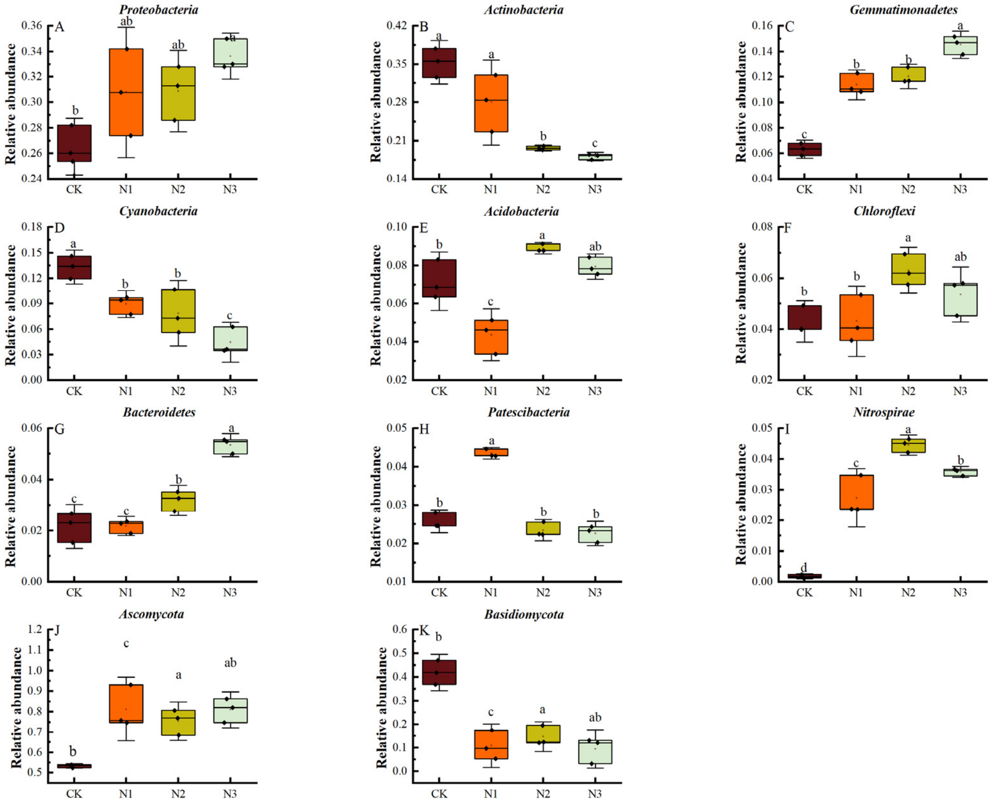

Figure S3. Analysis of differences in the relative abundance of dominant bacterial phyla and fungal phyla. CK, no fertilizer control; N1, 280 N kg ha^{-1}; N2, 560 N kg ha^{-1}; N3, 840 N kg ha^{-1}. Different letters indicate significant differences among treatments (Duncan's post hoc test, *p* < 0.05).
